# The relationships between urinary glycosaminoglycan levels and phenotypes of mucopolysaccharidoses

**DOI:** 10.1002/mgg3.471

**Published:** 2018-09-16

**Authors:** Hsiang‐Yu Lin, Chung‐Lin Lee, Yun‐Ting Lo, Tuan‐Jen Wang, Sung‐Fa Huang, Tzu‐Lin Chen, Yu‐Shan Wang, Dau‐Ming Niu, Chih‐Kuang Chuang, Shuan‐Pei Lin

**Affiliations:** ^1^ Department of Medicine Mackay Medical College New Taipei City Taiwan; ^2^ Department of Pediatrics Mackay Memorial Hospital Taipei Taiwan; ^3^ Department of Medical Research Mackay Memorial Hospital Taipei Taiwan; ^4^ Mackay Junior College of Medicine, Nursing and Management Taipei Taiwan; ^5^ Department of Medical Research China Medical University Hospital, China Medical University Taichung Taiwan; ^6^ Department of Laboratory Medicine Mackay Memorial Hospital Taipei Taiwan; ^7^ Department of Pediatrics Taipei Veterans General Hospital Taipei Taiwan; ^8^ College of Medicine Fu‐Jen Catholic University Taipei Taiwan; ^9^ Department of Infant and Child Care National Taipei University of Nursing and Health Sciences Taipei Taiwan

**Keywords:** dermatan sulfate, glycosaminoglycans, heparan sulfate, keratan sulfate, mucopolysaccharidosis, tandem mass spectrometry

## Abstract

**Background:**

The aim of this study was to use the liquid chromatography/tandem mass spectrometry (LC‐MS/MS) method to quantitate levels of three urinary glycosaminoglycans (GAGs; dermatan sulfate [DS], heparan sulfate [HS], and keratan sulfate [KS]) to help make a correct diagnosis of mucopolysaccharidosis (MPS).

**Methods:**

We analyzed the relationships between phenotypes and levels of urinary GAGs of 79 patients with different types of MPS.

**Results:**

The patients with mental retardation (*n* = 21) had significantly higher levels of HS than those without mental retardation (*n* = 58; 328.8 vs. 3.2 μg/ml, *p* < 0.001). The DS levels in the patients with hernia, hepatosplenomegaly, claw hands, coarse face, valvular heart disease, and joint stiffness were higher than those without. Twenty patients received enzyme replacement therapy (ERT) for 1–12.3 years. After ERT, the KS level decreased by 90% in the patients with MPS IVA compared to a 31% decrease in the change of dimethylmethylene blue (DMB) ratio. The DS level decreased by 79% after ERT in the patients with MPS VI compared to a 66% decrease in the change of DMB ratio.

**Conclusions:**

The measurement of GAG fractionation biomarkers using the LC‐MS/MS method is a more sensitive and reliable tool than the DMB ratio for MPS high‐risk screening, diagnosis, subclass identification, and monitoring the efficacy of ERT.

**Abbreviations**
2‐D EP: two‐dimensional electrophoresis
CS: chondroitin sulfate
DMB: dimethylmethylene blue
DS: dermatan sulfate
ERT: enzyme replacement therapy
GAGs: glycosaminoglycans
HS: heparan sulfate
KS: keratan sulfate
LC‐MS/MS: liquid chromatography/tandem mass spectrometry
MPS: mucopolysaccharidosis

## 1 INTRODUCTION

The mucopolysaccharidoses (MPSs) are a group of lysosomal storage disorders caused by the deficiency of specific enzymes that catalyze the stepwise degradation of glycosaminoglycans (GAGs). There are 11 known enzymes involved in the catabolism of dermatan sulfate (DS), heparan sulfate (HS), keratan sulfate (KS), chondroitin sulfate (CS), and hyaluronic acid. Specific enzyme deficiency blocks GAG degradation, which can cause severe organ and tissue dysfunction due to the accumulation of GAGs in cells. The clinical presentations of MPS are chronic and progressive, and the severity and prognosis vary among the different types with a wide spectrum of clinical severity (Chuang & Lin, 2007; Neufield & Muenzer, 2001). The clinical manifestations in these patients include coarse facial features, organomegaly, developmental delay, short stature, and skeletal deformities (dysostosis multiplex). In addition, vision and hearing conditions, airway obstruction, abnormal cardiovascular function, pulmonary function, bone mineral density, and joint mobility have also been reported (Lin et al., 2013; Lin et al., 2014; Lin et al., 2014; Lin, Chen, Lin, et al., 2010; Lin, Lin, Chuang, Lin, & Chen, 2014; Muenzer, 2004, 2011). The clinical manifestations may present from early to late childhood or even in early adulthood depending on the severity and type of MPS. Respiratory failure, cardiac failure, and recurrent respiratory infections usually occur before 10 years of age in patients with the severe forms (Jones et al., 2009; Lin, Chuang, Chen, Chiu, et al., 2014; Lin, Chuang, Huang, et al., 2016).

The clinical symptoms of the various types of MPS disorders can be classified into three groups according to the type of GAG accumulation. The “Visceral” group is caused by DS and includes patients with MPS I, II, VI, and VII presenting with coarse facial features, corneal clouding, adenotonsillar hypertrophy, hearing loss, upper airway obstruction, heart disease, hepatosplenomegaly, short stature, joint stiffness, and skeletal deformities (Golda, Jurecka, & Tylki‐Szymanska, 2012; Harmatz & Shediac, 2017). The “Neurodegenerative” group is caused by HS and includes patients with MPS IIIA, B, C, and D, MPS I (Hurler syndrome), and the severe form of MPS II presenting with cognitive decline, hyperactivity, and behavioral disturbances (Coutinho, Lacerda, & Alves, 2012; Tomatsu et al., 2005). The “Skeletal” group is caused by KS and includes patients with MPS IV presenting with joint laxity, genu valgum, odontoid hypoplasia, extreme short stature, and skeletal dysplasia (Hendriksz et al., 2015). The amounts of the affected GAG‐derived disaccharides in the different types of MPS have been reported to be associated with the clinical manifestations, severity, and onset of MPS disorders (Auray‐Blais et al., 2016; Mashima, Sakai, Tanaka, Kosuga, & Okuyama, 2016; Tomatsu et al., 2014).

An accurate diagnosis is traditionally made through three sequential examinations: quantification of urinary GAGs, two‐dimensional electrophoresis (2‐D EP) qualitative analysis, and leukocyte enzymatic activity assay (Chuang, Lin, & Chung, 2001; Chuang, Lin, Lee, & Wang, 2002). Urinary GAG quantitative analysis can be used as a diagnostic screening tool for MPS disorders; however, it cannot be used to identify a specific MPS disease. The dimethylmethylene blue (DMB) spectrophotometric method is widely used in most biochemical genetics laboratories; however, it is determined using non‐specific total GAG analysis which can lead to both false‐positive and false‐negative results, mainly in patients with MPS III and IV (Chuang et al., 2002; Piraud, Maire, & Mathieu, 1993). 2‐D EP is the most commonly used method to help determine specific MPS diseases; however, it is time‐consuming and the interpretation is ambiguous and subjective, making the diagnosis unreliable. To overcome the limitations of these first‐line MPS screening methods, the liquid chromatography/tandem mass spectrometry (LC‐MS/MS) method has been used to determine MPS subgroups, and it has been demonstrated to be a valuable and important method (Auray‐Blais et al., 2011, 2016; Chuang et al., 2014; Mashima et al., 2016).

The main treatments for MPS disorders include enzyme replacement therapy (ERT) and hematopoietic stem cell transplantation (Giugliani, Federhen, & Vairo, 2016). ERT is currently available for MPS I, II, IVA, VI, and VII, and it has been shown to improve endurance, joint mobility, and lung function in patients with MPS (Harmatz et al., 2018; Harmatz, Giugliani, & Schwartz, 2008, 2006; Hendriksz et al., 2014, 2016; Lin et al., 2010, 2005 ; Lin, Chuang, Wang, et al., 2016; Muenzer et al., 2006; Wraith, Clarke, & Beck, 2004). Although these treatments cannot cure the diseases, they can improve or alleviate the progression of the natural course. Several emerging therapies for MPS disorders are currently under development, including substrate reduction therapy and gene therapy (Giugliani et al., 2016).

The purpose of this study was to verify the LC‐MS/MS method for the quantitation of three urinary GAGs (DS, HS, and KS) to help make a correct diagnosis of MPS compared with the traditional DMB spectrophotometric method. In addition, we analyzed the relationships between phenotypes and levels of urinary GAGs, and evaluated the efficacy of ERT.

## 2 MATERIALS AND METHODS

### 2.1 Ethical compliance

The study protocol was approved by the Ethics Committee of Mackay Memorial Hospital, and written informed consent was provided by a parent of the children and from the patients themselves if they were over 18 years of age.

### 2.2 Study population

Seventy‐nine patients diagnosed with different types of MPS (type I [*n* = 14], II mild form [*n* = 14], II severe form [*n* = 9], III [*n* = 11], IV [*n* = 23], and VI [*n* = 8]; 57 males and 22 females; age range: 0.7–49.3 years) were enrolled from January 2013 to December 2017 at Mackay Memorial Hospital, Taipei, Taiwan. The levels of three urinary GAGs (DS, HS, and KS) were evaluated using the LC‐MS/MS method, and the non‐specific total level of urinary GAGs (DMB/creatinine ratio) was evaluated using the DMB spectrophotometric method as previously described (Auray‐Blais et al., 2011, 2016 ; Chuang et al., 2001, 2002, 2014 ; Chuang, Lin, & Lin, 2017; Kubaski et al., 2017; Martell et al., 2011; Oguma, Tomatsu, & Okazaki, 2007). The diagnosis of the type of MPS was confirmed by specific enzyme activity assays in serum, leukocytes and/or skin fibroblasts, and/or identification of a pathogenic mutation. For patients with MPS II, the severe form was defined according to the presence of cognitive impairment compared with the mild form (without cognitive impairment). None of the patients had received ERT or hematopoietic stem cell transplantation before entering this study. The levels of urinary GAGs of the 20 patients with MPS I, II, IVA, and VI who subsequently received ERT were also recorded. The levels of urinary DS, HS, and KS, and DMB ratios were checked at baseline and after ERT. All samples were stored at −20°C until analysis.

### 2.3 Normal reference values

Normal reference values of the three urinary GAGs (DS, HS, and KS) were established using the LC‐MS/MS method, and the non‐specific total level of urinary GAGs (DMB/creatinine ratio) was established using the DMB spectrophotometric method after the analysis of 221 healthy control urine samples (age range: 1 month to 49 years, median age: 9.1 years).

### 2.4 Clinical assessments

The demographic data including gender, type of MPS, and age at the start of ERT and duration of ERT, as well as the existence of clinical manifestations including mental retardation, hernia, hepatosplenomegaly, claw hands, coarse face, adenotonsillar hypertrophy, valvular heart disease, joint stiffness, and hypermobile joints, were recorded for each patient.

### 2.5 Statistical analysis

Descriptive statistics including means, standard deviations, and percentage changes in urinary DS, HS, and KS, and DMB ratio before and after ERT were analyzed. The relationships between the levels of the three urinary GAGs and the existence of each clinical manifestation in the patients with MPS were tested using Pearson's chi‐squared test. Two‐tailed *p* values were computed. SPSS version 11.5 (SPSS Inc., Chicago, IL, USA) was used for calculations, and differences were considered to be statistically significant when the *p* value was <0.05.

## 3 RESULTS

### 3.1 Normal reference values

Normal reference values were established after the analysis of 221 healthy control urine samples. We divided the data into five age ranges as follows: <1, 1–3, 4–9, 10–17, and >18 years of age. The normal reference values of each age‐group were determined as the mean plus two standard deviations. The normal reference ranges of DS, HS, and KS were similar among the five age‐groups. However, the normal reference ranges of DMB ratio decreased with the increasing age of the normal controls (Table 1).

**Table 1:** Normal reference values for urinary DS, HS, KS using the liquid chromatography/tandem mass spectrometry method and DMB ratio using the DMB spectrophotometric method according to different age‐groups

Age (years)	*N*	DS (μg/ml)	HS (μg/ml)	KS (μg/ml)	DMB ratio (mg/mmol creatinine)
<1	38	<0.32	<0.57	<8.34	<69.15
1–3	45	<0.36	<0.32	<6.64	<58.82
4–9	32	<0.28	<0.11	<6.98	<18.20
10–17	9	<0.28	<0.28	<8.86	<16.81
>18	97	<0.54	<0.49	<1.69	<12.75
All	221	<0.43	<0.46	<6.03	Not defined

Note

DS: dermatan sulfate; HS: heparan sulfate; KS: keratan sulfate; DMB: dimethylmethylene blue.

### 3.2 Urinary DS, HS, and KS, and DMB ratio in untreated patients with different types of MPS

**Figure 1:**
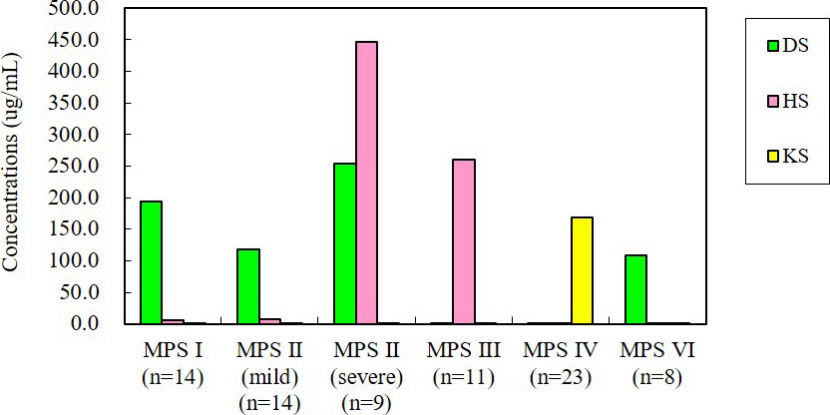
The mean levels of DS, HS, and KS in different types of MPS (*n* = 79). DS: dermatan sulfate; HS: heparan sulfate; KS: keratan sulfate; MPS: mucopolysaccharidosis

The highest mean level of DS (reference <0.43 μg/ml) was noted in the patients with the severe form of MPS II (253.8 μg/ml), followed by MPS I (194.6 μg/ml), the mild form of MPS II (118.0 μg/ml), and MPS VI (108.9 μg/ml). The highest mean level of HS (reference <0.46 μg/ml) was found in the patients with the severe form of MPS II (446.6 μg/ml), followed by MPS III (260.7 μg/ml), the mild form of MPS II (7.3 μg/ml), and MPS I (6.2 μg/ml). The mean level of KS (reference <6.03 μg/ml) in the patients with MPS IV was 168.0 μg/ml (Table 2 and Figure 1).

**Table 2:** The levels of urinary DS, HS, and KS, and DMB ratio in different types of MPS (*n* = 79)

Type	*N*	Age (years)	DS (μg/ml)	HS (μg/ml)	KS (μg/ml)	DMB ratio (mg/mmol creatinine)
MPS I	14	16 (0.7–34.9)	194.6 (62.0–414.6)	6.2 (0.11–26.8)	1.3 (0–3.0)	53.1 (13.6–159.5)
MPS II (mild)	14	18.1 (1.4–37.8)	118.0 (2.7–520.3)	7.3 (0–42.7)	1.2 (0–5.54)	51.6 (10.3–101.4)
MPS II (severe)	9	9.1 (0.9–21.5)	253.8 (37.5–1,214.8)	446.6 (43.4–1654.4)	1.0 (0–1.62)	85.6 (24.8–185.4)
MPS III	11	7.4 (3.2–17.1)	0.4 (0–0.9)	260.7 (48.6–999.6)	0.8 (0–3.6)	46.2 (8.6–101.0)
MPS IV	23	15.1 (1.4–29.4)	0.2 (0–1.3)	0.1 (0–0.35)	168.0 (10.54–1,377.5)	16.7 (0–30.4)
MPS VI	8	11.9 (4.4–21.2)	108.9 (17.0–502.3)	1.2 (0–3.2)	0.6 (0–1.88)	37.0 (21.4–64.9)

Notes

Data are mean (range).

DS: dermatan sulfate; HS: heparan sulfate; KS: keratan sulfate; DMB: dimethylmethylene blue; MPS: mucopolysaccharidosis.

### 3.3 False‐negative rate

False negatives were defined as patients with MPS with normal results of urinary DMB ratio or urinary DS, HS, and KS. The false‐negative rate of the urinary DMB ratio using the spectrophotometric method for all 79 patients was 24%. Among the different types of MPS, MPS IV had the significantly highest false‐negative rate by this method (15/23, 65%), followed by MPS III (2/11, 18%) and the mild form of MPS II (2/14, 14%). However, there were no cases of false‐negative results of urinary DS, HS, and KS using the LC‐MS/MS method (Table 3).

**Table 3:** Comparisons of false‐negative rates between urinary DMB ratio using the DMB spectrophotometric method and quantification of DS, HS, and KS using the liquid chromatography/tandem mass spectrometry method

Type	*N*	Urinary DMB ratio: false negative (*N*)	Urinary DMB ratio: false negative (%)	Urinary DS, HS, KS: false negative (*N*)	Urinary DS, HS, KS: false negative (%)
MPS I	14	0	0	0	0
MPS II (mild)	14	2	14	0	0
MPS II (severe)	9	0	0	0	0
MPS III	11	2	18	0	0
MPS IV	23	15	65	0	0
MPS VI	8	0	0	0	0
Total	79	19	24	0	0

Note

DMB: dimethylmethylene blue; DS: dermatan sulfate; HS: heparan sulfate; KS: keratan sulfate; MPS: mucopolysaccharidosis.

### 3.4 The relationships between clinical manifestations and the levels of urinary DS, HS, and KS, and DMB ratio

Among the 79 patients, those with mental retardation (*n* = 21) had significantly higher levels of HS than those without mental retardation (*n* = 58; 328.8 vs. 3.2, *p* < 0.001). However, the DS levels in the patients with hernia, hepatosplenomegaly, claw hands, coarse face, valvular heart disease, and joint stiffness were significantly higher than in those without these clinical manifestations (*n* = 68, *p* < 0.05). In addition, the KS levels in the patients with hypermobile joints were significantly higher than in those without hypermobile joints (*n* = 68, *p* < 0.001; Table 4).

**Table 4:** Relationships between clinical manifestations and levels of urinary DS, HS, and KS, and DMB ratio in 79 patients with MPS

Clinical manifestations	With or without	*N*	DS (μg/ml)	*p* value	HS (μg/ml)	*p* value	KS (μg/ml)	*p* value	DMB ratio (mg/mmol creatinine)	*p* value
Mental retardation	With	21	116.2	0.530	328.8	<0.001	0.9	0.072	65.4	0.028
	Without	58	87.9		3.2		94.7		39.6	
Hernia	With	43	159.9	0.001	89.6	0.572	14.3	0.058	52.0	0.085
	Without	25	11.0		129.0		97.0		35.5	
Hepatosplenomegaly	With	45	143.6	0.016	132.2	0.241	3.5	0.005	51.0	0.129
	Without	23	30.1		49.2		125.4		36.1	
Claw hands	With	48	147.2	0.003	85.3	0.385	12.6	0.017	47.9	0.514
	Without	20	4.2		149.3		121.8		41.2	
Coarse face	With	56	127.6	0.030	110.6	0.675	21.1	0.015	46.0	0.964
	Without	12	0.3		73.7		154.8		45.5	
Adenotonsillar hypertrophy	With	45	131.3	0.105	68.8	0.138	38.7	0.693	47.3	0.691
	Without	23	54.1		173.3		56.5		43.3	
Valvular heart disease	With	55	128.6	0.030	95.4	0.633	23.8	0.030	45.1	0.543
	Without	12	0.2		138.0		144.4		38.3	
Joint stiffness	With	51	138.6	0.009	134.2	0.118	1.1	<0.001	51.0	0.057
	Without	17	4.8		13.9		175.5		30.7	
Hypermobile joints	With	13	0.2	0.022	0.1	0.129	208.2	<0.001	3.9	<0.001
	Without	55	130.0		128.7		6.0		51.4	

Note

DS: dermatan sulfate; HS: heparan sulfate; KS: keratan sulfate; DMB: dimethylmethylene blue; MPS: mucopolysaccharidosis.

### 3.5 The existence of each clinical manifestation among the different types of MPS

**Figure 2:**
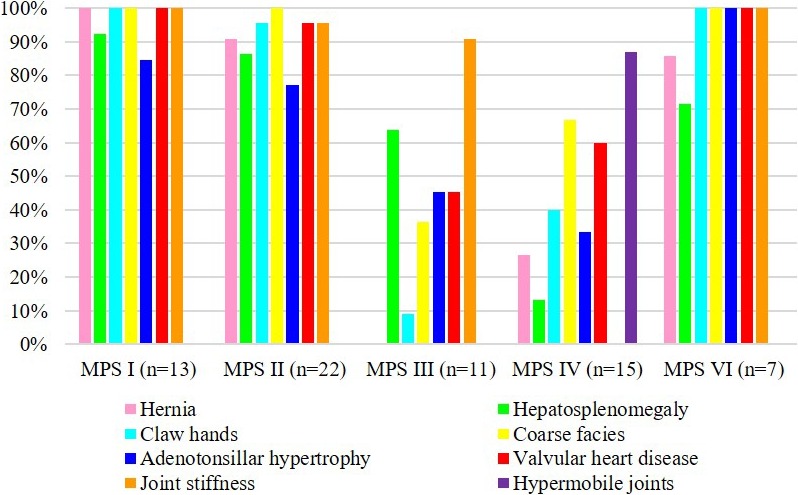
Clinical manifestations of 68 patients with different types of mucopolysaccharidosis (MPS)

Data were available on 68 patients with hernia, hepatosplenomegaly, claw hands, coarse face, adenotonsillar hypertrophy, valvular heart disease, joint stiffness, and hypermobile joints. Most of the patients with MPS I, II, and VI had hernia, hepatosplenomegaly, claw hands, coarse face, adenotonsillar hypertrophy, and valvular heart disease (71%–100%); however, fewer patients with MPS III and IV had these features (0%–67%). Eighty‐seven percent of the patients with MPS IV had hypermobile joints; however, none of the patients with MPS I, II, III, and VI had this feature. Over 90% of the patients with MPS I, II, III, and VI (91%–100%) had joint stiffness, compared with none of the patients with MPS IV. No patient with MPS III had hernia in this cohort (Figure 2).

### 3.6 The longitudinal follow‐up values of urinary DS, HS, and KS, and DMB ratio for the patients who received ERT for MPS

**Figure 3:**
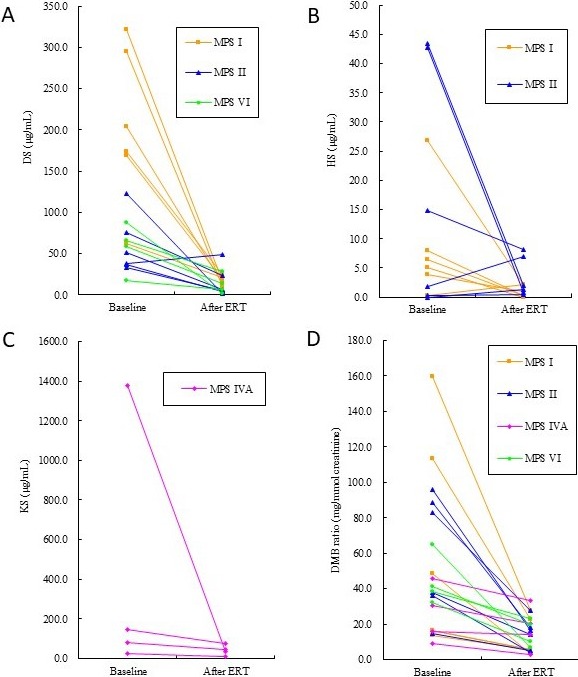
Changes in urinary DS (A), HS (B), and KS (C), and DMB ratio (D) of 20 patients with MPS I, II, IVA, or VI receiving ERT for 1–12.3 years. DS: dermatan sulfate; HS: heparan sulfate; KS: keratan sulfate; DMB: dimethylmethylene blue; MPS: mucopolysaccharidosis; ERT: enzyme replacement therapy

Twenty patients received ERT for 1–12.3 (mean ± standard deviation, 5.5 ± 3.5) years, including six patients with MPS I, six with MPS II, four with MPS IVA, and four with MPS VI. An average of 289 infusions of ERT was given. After ERT, the DMB ratio decreased by 81%, 76%, 31%, and 66% for MPS I, MPS II, MPS IVA, and MPS VI, respectively. The DS levels decreased by 91%, 75%, and 79% for MPS I, MPS II, and MPS VI, respectively, and the HS levels decreased by 88% and 81% for MPS I and MPS II, respectively. For MPS IVA, the KS level decreased by 90% after ERT compared with a decrease of only 31% in the change of DMB ratio (Table 5 and Figure 3).

**Table 5:** Changes in urinary DS, HS, and KS, and DMB ratio of 20 patients with MPS I, II, IVA, or VI receiving ERT for 1–12.3 years

Type	*N*	Age at start of ERT (y)	ERT duration (y)	ERT duration (wk)	DS (μg/ml)		Change (%)	HS (μg/ml)		Change (%)	KS (μg/ml)		Change (%)	DMB ratio (mg/mmol creatinine)		Change (%)
					Baseline	After ERT		Baseline	After ERT		Baseline	After ERT		Baseline	After ERT	
MPS I	6	18.4 (0.7– 34.9)	3.7 (2.0–8.3)	195 (104– 434)	204.3	17.4	−91%	8.4	1.0	−88%	–	–	–	61.3	11.8	−81%
MPS II	6	8.4 (3.2–17.5)	6.0 (1.0–9.3)	312 (52–486)	59.6	15.0	−75%	17.2	3.3	−81%	–	–	––	59.3	14.2	−76%
MPS IVA	4	11.7 (1.4–16.8)	3.9 (2.1–5.3)	201 (109– 277)	–	–	–	–	–	–	407.8	40.3	−90%	25.3	17.5	−31%
MPS VI	4	11.1 (7.6–16.7)	9.3 (4.0–12.3)	485 (210–640)	57.1	12.2	−79%	–	–	–	–	–	–	44.1	14.9	−66%

Note

Data are mean (range).

DS, dermatan sulfate; HS, heparan sulfate; KS, keratan sulfate; DMB, dimethylmethylene blue; MPS, mucopolysaccharidosis; ERT, enzyme replacement therapy.

## 4 DISCUSSION

To the best of our knowledge, this is the first report to describe the relationships between phenotypes of MPS and quantitative levels of urinary GAGs (DS, HS, and KS) using the LC‐MS/MS method in patients with different types of MPS in a single institution. In this study, every patient was assessed at our clinic by the same investigator (SPL) to minimize inter‐observer variation. Patients with mental retardation had higher levels of HS compared to those without mental retardation. In addition, the patients with hernia, hepatosplenomegaly, claw hands, coarse face, valvular heart disease, and joint stiffness had higher levels of DS compared to those without these conditions, and the patients with hypermobile joints had higher KS levels than those without hypermobile joints. Previous studies have reported that HS can lead to dysfunction of the central nervous system (Coutinho et al., 2012; Tomatsu et al., 2005), that DS may principally result in soft tissue storage and skeletal involvement (Golda et al., 2012; Harmatz & Shediac, 2017), and that KS may primarily cause skeletal dysplasia and non‐bone soft tissue (Hendriksz et al., 2015). Our results also demonstrated these findings according to the quantitative data of urinary GAGs (DS, HS, and KS) using the LC‐MS/MS method.

Auray‐Blais et al. (2016) reported the quantitation of urinary GAGs (DS, HS, and KS) using a reliable tandem mass spectrometry multiplex method. This method allowed for the differentiation of MPS types except for MPS I and MPS II which had the same GAG profile. Our results also showed that the LC‐MS/MS method was highly sensitive and specific to discriminate DS, HS, and KS, and that it could be used to differentiate different types of MPS. In other words, our results suggest that the LC‐MS/MS method could be used to identify the quantities of affected GAGs and that this could be used to determine the type of MPS. This is consistent with studies using the 2‐D EP method and leukocyte enzymatic assays. The LC‐MS/MS method appears to be a highly sensitive, specific, and reliable method to determine the type of MPS, with high throughput and automatic analysis.

The LC‐MS/MS method can also be used to differentiate the subtypes of MPS II, and the concentration of HS is the main factor used to distinguish the mild form of MPS II from the severe form. Mashima et al. (2016 reported that among patients who received ERT with confirmed elevation of antibody titers, the concentration of HS in the urine of patients with the severe form was higher than that in those with the mild form of MPS II. Consistently, the concentrations of HS detected in the samples of our patients with the severe form of MPS II were more than 60 times higher than those of the patients with the mild form (446.6 vs. 7.3 μg/ml). The concentrations of DS also varied among the patients with MPS I, II, and VI. The highest levels of DS were detected in the patients with the severe form of MPS II followed by MPS I, and they were significantly higher than in those with the mild form of MPS II and MPS VI.

Auray‐Blais et al. (2016) reported that the DMB spectrophotometric method may lead to false‐negative results, especially in MPS III and IV, due to aggregated formation by electrostatic interactions with collagen, glycoproteins, albumin, or other serum proteins which may then modify the physicochemical properties of GAGs (Piraud et al., 1993). In their study, the DMB spectrophotometric method missed the detection of MPS in 30% of their patients (7/23; one with MPS II, one with MPS III, four with MPS IVA, and one with MPS VI), confirming that this method is not reliable for screening MPS patients. Our results are consistent with theirs, and the false‐negative rate of the urinary DMB ratio using the spectrophotometric method for all 79 patients in our cohort was 24%. Among all types of MPS, MPS IV had the significantly highest false‐negative rate by this method (65%), followed by MPS III (18%) and the mild form of MPS II (14%). However, no case had a false‐negative result of urinary DS, HS, and KS by the LC‐MS/MS method.

MPS I and II have many similar clinical features. The severe forms of MPS I and II both include somatic and cognitive involvement, and their signs and symptoms include coarse facial features, vision and hearing loss, decreased pulmonary function, obstructive sleep apnea, recurrent respiratory infections, cardiac disease, hepatosplenomegaly, umbilical and inguinal hernias, dysostosis multiplex, communicating hydrocephalus, and spinal cord compression. MPS III presents with cognitive and neurological impairment as well as mild somatic involvement. MPS IV is characterized by skeletal dysplasia, joint hypermobility, ligamentous laxity, odontoid hypoplasia, and short stature. MPS VI manifests as a purely somatic disease similar to those seen with MPS I and II, with no cognitive involvement (Muenzer, 2004, 2011; Neufield et al., 2001). In the present study, 68 patients had available data on the presence of hernia, hepatosplenomegaly, claw hands, coarse face, adenotonsillar hypertrophy, valvular heart disease, joint stiffness, and hypermobile joints. Most of the patients with MPS I, II, and VI had hernia, hepatosplenomegaly, claw hands, coarse face, adenotonsillar hypertrophy, and valvular heart disease; however, fewer patients with MPS III and IV had these features. Eighty‐seven percent of the patients with MPS IV had hypermobile joints; however, none of the patients with MPS I, II, III, and VI had this feature. In addition, over 90% of the patients with MPS I, II, III, and VI had joint stiffness, compared to none of the patients with MPS IV. None of the patients with MPS III had a hernia in this cohort.

Previous studies have shown that ERT for MPS I, II, IVA, and VI can lead to good clearance of urine GAGs and significantly improve a wide range of physiological activities and quality of life (Chuang et al., 2017; Harmatz, Giugliani, & Schwartz, 2008, 2006 ; Hendriksz et al., 2014, 2016 ; Lin et al., 2005; Lin, Chen, Chuang, et al., 2010; Lin, Chuang, Wang, et al., 2016; Muenzer et al., 2006; Ru et al., 2013; Wraith et al., 2004). The DMB ratio can provide useful information to evaluate the effectiveness of ERT from a biochemical point of view; however, it cannot directly reflect the efficacy of DS, HS, or KS degradation after ERT. Only a few studies have described changes in urine DS, HS, or KS after ERT for MPS I, II, IVA, and VI (Chuang et al., 2017, 2014 ; Ru et al., 2013). In the present study, the KS level decreased by 90% after ERT for MPS IVA compared with only a 31% decrease in the change of DMB ratio. In addition, the DS level decreased by 79% after ERT for MPS VI compared to only a 66% decrease in the change of DMB ratio, and the HS level decreased by 88% and 81% after ERT compared to decreases of 81% and 76% in the change of DMB ratio for MPS I and II, respectively. In other words, our data showed greater reductions in the concentrations of DS, HS, or KS after ERT using the LC‐MS/MS method than the DMB ratio using the spectrophotometric method. They therefore seem to be more sensitive biomarkers than the traditional DMB ratio in evaluating the efficacy of ERT for MPS.

### 4.1 Limitations

As a retrospective study, not all clinical data were available for all of our subjects. For the measurement of ERT efficacy, data were only available for quantification at baseline and one post‐infusion measurement. Some patients had more infusions, and others had less. The small sample size of each type of MPS reflects the rare nature of this genetic disorder. Meanwhile, the variations in genotypes and the degree of disease severity were quite wide, as was the age range. Further studies with larger cohorts and a longer follow‐up period are warranted.

## 5 CONCLUSIONS

The levels of DS, HS, and KS were associated with specific phenotypes of MPS in our cohort. The patients with mental retardation had higher levels of HS compared to those without mental retardation, and the patients with hernia, hepatosplenomegaly, claw hands, coarse face, valvular heart disease, and joint stiffness had higher levels of DS compared to those without these features. In addition, the patients with hypermobile joints had higher levels of KS than those without hypermobile joints. The patients with the severe form of MPS II had significantly higher HS levels than those with the mild form of MPS II. The measurement of GAG fractionation biomarkers using the LC‐MS/MS method is an accurate and reliable tool to simultaneously quantify urinary levels of DS, HS, and KS, and these biomarkers are more sensitive than the traditional DMB ratio using the spectrophotometric method to diagnose MPS, screen high‐risk populations, identify subgroups, and evaluate the efficacy of ERT.

## CONFLICT OF INTEREST

The authors declare that they have no competing interests.

## AUTHORS' CONTRIBUTIONS

HYL performed acquisition, statistical analysis and interpretation of data, and drafted the manuscript. SPL and CKC participated in the design of the study, interpretation of the data and helped to draft the manuscript. CLL, YTL, TJW, SFH, TLC, and YSW performed biochemical analyses and revised the manuscript. DMN was responsible for patient screening and revised the manuscript. All authors read and accepted the manuscript.
